# Cell-free fat extract promotes tissue regeneration in a tissue expansion model

**DOI:** 10.1186/s13287-020-1564-7

**Published:** 2020-02-04

**Authors:** Mingwu Deng, Xiangsheng Wang, Ziyou Yu, Yizuo Cai, Wei Liu, Guangdong Zhou, Xiansong Wang, Zheyuan Yu, Wei Li, Wen Jie Zhang

**Affiliations:** Department of Plastic and Reconstructive Surgery, Shanghai 9th People’s Hospital, Shanghai Jiao Tong University School of Medicine, Shanghai Key Laboratory of Tissue Engineering, National Tissue Engineering Center of China, 639 ZhiZaoJu Road, Shanghai, 200011 China

**Keywords:** Cell-free fat extract, Tissue expansion, Pro-angiogenic, Pro-proliferative

## Abstract

**Background:**

Tissue expansion techniques play an important role in plastic surgery. How to improve the quality of the expanded skin and shorten the expansion period are still worth investigating. Our previous studies found that a cell-free fat extract (CEFFE) possessed pro-angiogenic and pro-proliferative activities. However, the role of CEFFE on tissue expansion has remained unclear. The purpose of this study was to evaluate the effect of CEFFE on tissue expansion.

**Methods:**

A rat tissue expansion model was used. Animals were treated with CEFFE by subcutaneous injection. After 4 weeks of tissue expansion, the skin necrosis and retraction rates were evaluated, the thicknesses of the epidermis and dermis were determined by histological analyses, blood vessel density was measured by anti-CD31 staining, cell proliferation was assessed by proliferating cell nuclear antigen staining, and the expression of specific proteins was evaluated by western blot analyses. In addition, the effects of CEFFE on the proliferation and cell cycle of cultured HaCaT cells were evaluated in vitro.

**Results:**

CEFFE treatment significantly decreased the necrosis rate and retraction of the expanded skin. The thickness of the epidermal and dermal layers was higher in CEFFE-treated compared to untreated skin. The density of blood vessels and cell proliferation in the epidermis of the expanded skin was improved by CEFFE treatment. In addition, CEFFE treatment significantly increased the expression of the vascular endothelial growth factor receptor, epidermal growth factor receptor, collagen type 1, and collagen type 3. CEFFE also increased the proliferation of HaCaT cells in culture.

**Conclusions:**

CEFFE improves the quality of the expanded skin by promoting angiogenesis and cell proliferation. It could be potentially used clinically for augmenting tissue expansion.

## Background

Repairing skin defects, such as burn deformities and large scar excision areas, is a major clinical challenge [1, 2]. Currently, the most commonly used method to repair such defects is skin grafting [3]. However, this method has the disadvantages of few donor sources, skin texture, and color differences between the transplanted and surrounding skin, and creating another wound at the donor site [3, 4].

Tissue expansion is a technique proposed by Neumann in 1957. The technique involves implanting a soft tissue expander under the body surface, which stimulates skin proliferation by mechanical stretching, thus obtaining an extra skin [5–7]. Currently, it is one of the most important techniques in plastic surgery, playing an important role in repairing scalp defects, treating scar tissue, ear reconstruction, breast reconstruction, and other fields [8–11]. However, there are shortcomings in tissue expansion such as the long amplification cycle period and complications [12]. Many methods have been used to obtain sufficient skin and reduce the amplification cycle time of tissue expansion. For example, rapid expansion can shorten the expansion time. However, there are disadvantages to this approach such as a high necrosis rate due to an insufficient blood supply, and a high retraction rate due to the expanded tissue consisting, in large part, of stretched preexisting tissue [13].

To help the skin expand, many studies have applied drugs, such as anti-contractile agents and dimethyl sulfoxide. However, these drugs can elicit additional complications [14, 15]. Recently, stem cell therapy has been tested in tissue expansion. Adipose-derived stem cells are rich in adipose tissue and easy to obtain and have thus received wide attention from researchers. Studies have shown that adipose-derived stem cells improve skin expansion efficiency by secreting growth factors such as vascular endothelial growth factor (VEGF) and epidermal growth factor (EGF) [16, 17]. However, immunity and tumorigenicity of stem cells restrict their application [18]. Adipose tissue plays an important role in tissue regeneration. On the one hand, adipose tissue contains a variety of cells that can secrete a large number of growth factors related to tissue regeneration [19]. It has been reported that the conditioned medium from adipose tissue could promote wound healing [20]. Studies also showed that conditioned medium from cultured adipose-derived stem cells have the ability to promote tissue regeneration [21, 22]. On the other hand, acellular adipose tissue as an important biomaterial, could also promote soft tissue regeneration [23, 24].

Our previous studies demonstrated that, after emulsification and centrifugation of adipose tissue, we could efficiently remove the oil droplets, cell debris, and extracellular matrix and obtain a cell-free liquid fraction that was enriched with growth factors. We named this a cell-free fat extract (CEFFE). CEFFE contains large amounts of VEGF, EGF, and other growth factors, which can promote angiogenesis [25]. In addition, it can promote cell proliferation and collagen secretion [26]. Based on the biological of activities of CEFFE, we speculated that it might be useful in the skin expansion. In this study, a rat skin expansion model was used to explore the role and mechanism of CEFFE in skin expansion.

## Methods

### CEFFE preparation

CEFFE was obtained as described previously [25]. Briefly, with the patient’s informed consent, adipose tissue was obtained after liposuction, then washed with saline and centrifuged to remove water and oil. We obtained CEFFE after mechanical emulsification and centrifugation. CEFFE was stored at – 80 °C. The protein concentration of CEFFE was determined with a bicinchoninic acid assay kit (Thermo Fisher Scientific, Waltham, MA, USA).

### Skin expansion model and treatment groups

All animal and surgical procedures are approved by the animal experimental center of Shanghai Jiao Tong University School of Medicine. Four-week-old Wistar female rats were divided into three groups: control (*n* = 12), CEFFE^Low^ (*n* = 8), and CEFFE^High^ (*n* = 8). The skin expansion model was performed as described previously with some modifications [16]. After implantation of the tissue expander, rats in the CEFFE^Low^ and CEFFE^High^ groups were injected subcutaneously with 300 or 600 μg of total CEFFE protein, respectively. Control animals did not receive any treatment. From the second day after implantation of the skin expander and CEFFE treatment, 5-mL physiological saline was injected into the expander once every other day for a total of 4 weeks. After 4 weeks, the skin tissue was observed and sampled.

### Retraction rate assessment

At the end of the 4-week expansion, a 3 × 3 cm^2^ section was drawn in the middle of the expanded skin. The skin was then cut in a U shape at the base of the expansion and laid flat. We measured the length and width after contraction and calculated the area. The formula for calculating the retraction percentage was retraction percentage = (9 - area after retraction)/9 × 100.

### Histological analyses

Skin samples were fixed with 4% paraformaldehyde, embedded in paraffin, and cut into 5-μm sections. Hematoxylin and eosin (HE), and Masson’s trichrome staining were performed. The epidermal and dermal thickness of each sample was measured using Image-Pro Plus 6 software (Media Cybernetics, Rockville, MD, USA).

### Immunohistochemical staining

To observe blood vessels, samples were incubated with a mouse anti-CD31 antibody, then with a horseradish peroxidase-conjugated goat anti-mouse antibody. The capillary density was calculated from five randomly selected fields from each sample. To observe cell proliferation, proliferating cell nuclear antigen (PCNA) staining was performed. The PCNA-positive cell number was calculated from five randomly selected fields from each sample.

### Western blot analyses

Western blot analyses were performed according to a standard protocol. The expression of VEGF and its receptor; VEGFR; EGF and its receptor; EGFR; basic fibroblast growth factor (bFGF); collagen type 1 (COL-1); collagen type 3 (COL-3); matrix metalloproteinase (MMP)-1, − 3, and – 9; and tissue inhibitor of metalloproteinases 1 (TIMP-1) was measured using mouse antibodies from Abcam (Cambridge, UK). Then, the membranes were incubated with a horseradish peroxidase-conjugated goat anti-mouse secondary antibody. β-actin was used as the loading control. Protein bands were visualized with enhanced chemiluminescence (Pierce, Rockford, IL, USA).

### Cell culture

The human keratinocyte HaCaT cell line was obtained from the American Type Culture Collection (Manassas, VA, USA). Cells were cultured in Dulbecco’s modified Eagle’s medium (Thermo Fisher Scientific) containing 10% fetal bovine serum (GE Healthcare Life Sciences, Logan, UT, USA), 100 U/mL penicillin, and 100 μg/mL streptomycin and placed in a humidified incubator containing 5% CO_2_.

### Cell proliferation assay

HaCaT cells were co-cultured with different concentrations of CEFFE (50, 250, and 500 μg/mL) for 72 h. Cell proliferation was detected with the Cell Counting Kit-8 assay (Beyotime Institute of Biotechnology, Haimen, China). Control cells were cultured without CEFFE treatment. Results are shown as percentages relative to the control group.

### Cell cycle analysis

HaCaT cells were co-cultured with different concentrations of CEFFE (50, 250, and 500 μg/mL), and the cell cycle analysis was performed 48 h later. Cultured cells were collected and fixed overnight with 70% ethanol, then incubated with RNase A (Merck KGaA, Darmstadt, Germany) and propidium iodide (Sigma-Aldrich, St. Louis, MO, USA). Cells were analyzed by flow cytometry (Beckman-Coulter, Brea, CA, USA) with Kaluza Analysis Software v.2.0.

### Statistical analyses

All data are expressed as means ± standard deviation. SPSS 19 software (IBM, Chicago, IL, USA) was used to conduct a one-way analysis of variance followed by Tukey’s post hoc test. *P* < 0.05 indicates a significant difference.

## Results

### CEFFE treatment reduced necrosis and retraction during rapid expansion

**Fig. 1:**
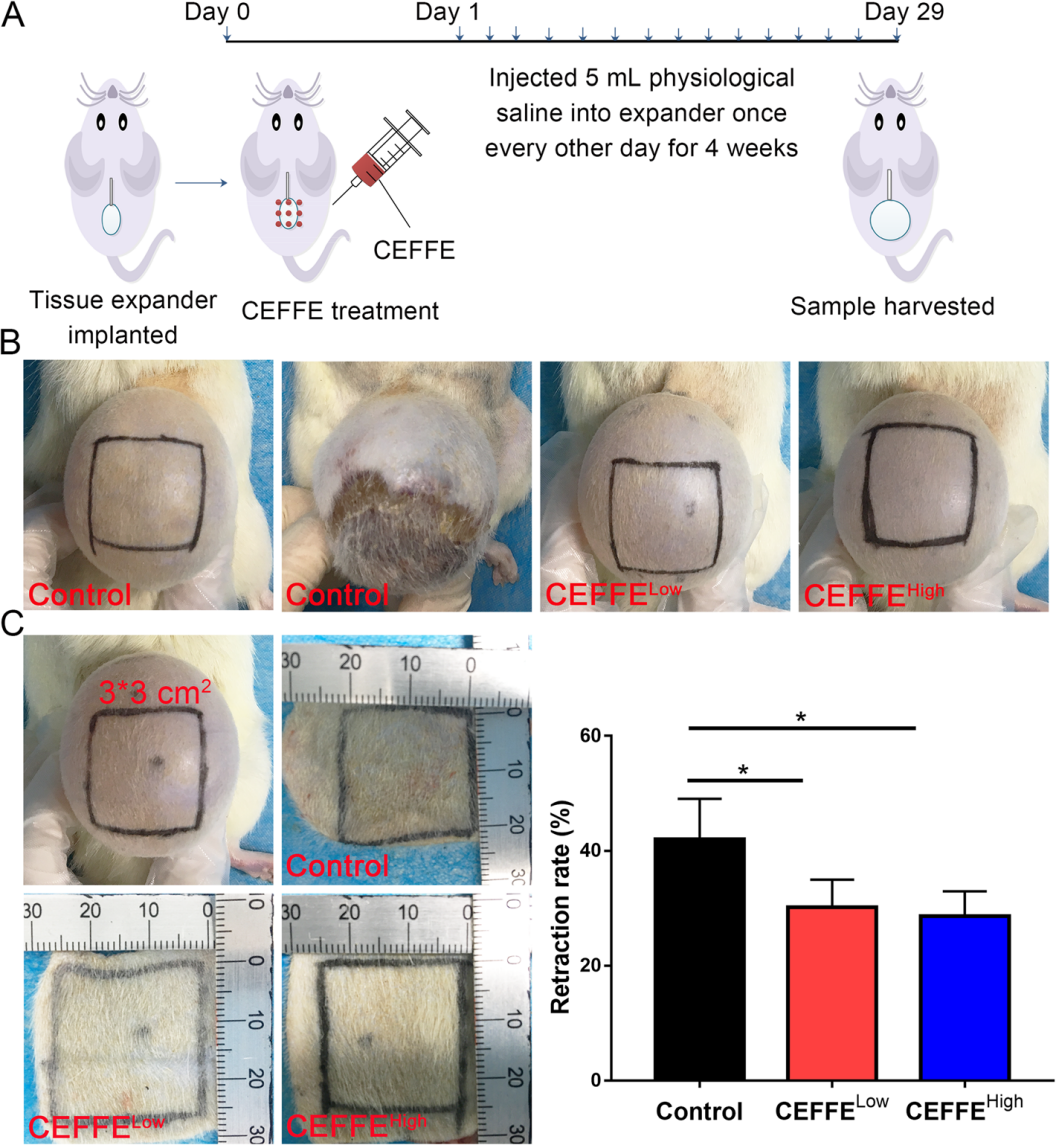
Flowchart of the experimental timeline and macroscopic observation of tissue after expansion. **a** The tissue expander is implanted subcutaneously and treated with cell-free fat extract (CEFFE) on day 0. From days 1 to 29, 5 mL of physiological saline was injected into the expander every other day for a total of 4 weeks. Then, the skin tissue was sampled and observed. **b** Macroscopic observation of the expanded tissue. Necrosis is present in the control tissue. **c** Retraction rate assessment of expanded tissue. A lower retraction rate is observed in the CEFFE-treated tissue. Data are expressed as means ± SD. ∗*p* < 0.05

After expansion, four of 12 untreated rats exhibited necrosis, a rate of about 33%. In contrast, in the CEFFE treatment groups, no necrosis was observed. Gross observation of the expanded tissue is shown in Fig. 1 and Table 1. The retraction rate in the control group was 42.0 ± 7.1%, in the CEFFE^Low^ group 28.7 ± 4.3%, and in the CEFFE^High^ group 30.2 ± 4.8%. The retraction rate following CEFFE treatment was significantly lower than that of the control rats. There was no significant difference between the CEFFE^Low^ and CEFFE^High^ groups. Gross observations indicated that CEFFE treatment effectively protected the expanded skin from necrosis.

**Table 1:** Necrosis rate of expanded tissue in different treatment groups

	Necrosis case	Necrosis rate
Control	4/12	33.33%
CEFFE^Low^	0/8	0
CEFFE^High^	0/8	0

### CEFFE treatment increased epidermal and dermal thickness, and collagen deposition in the expanded tissue

**Fig. 2:**
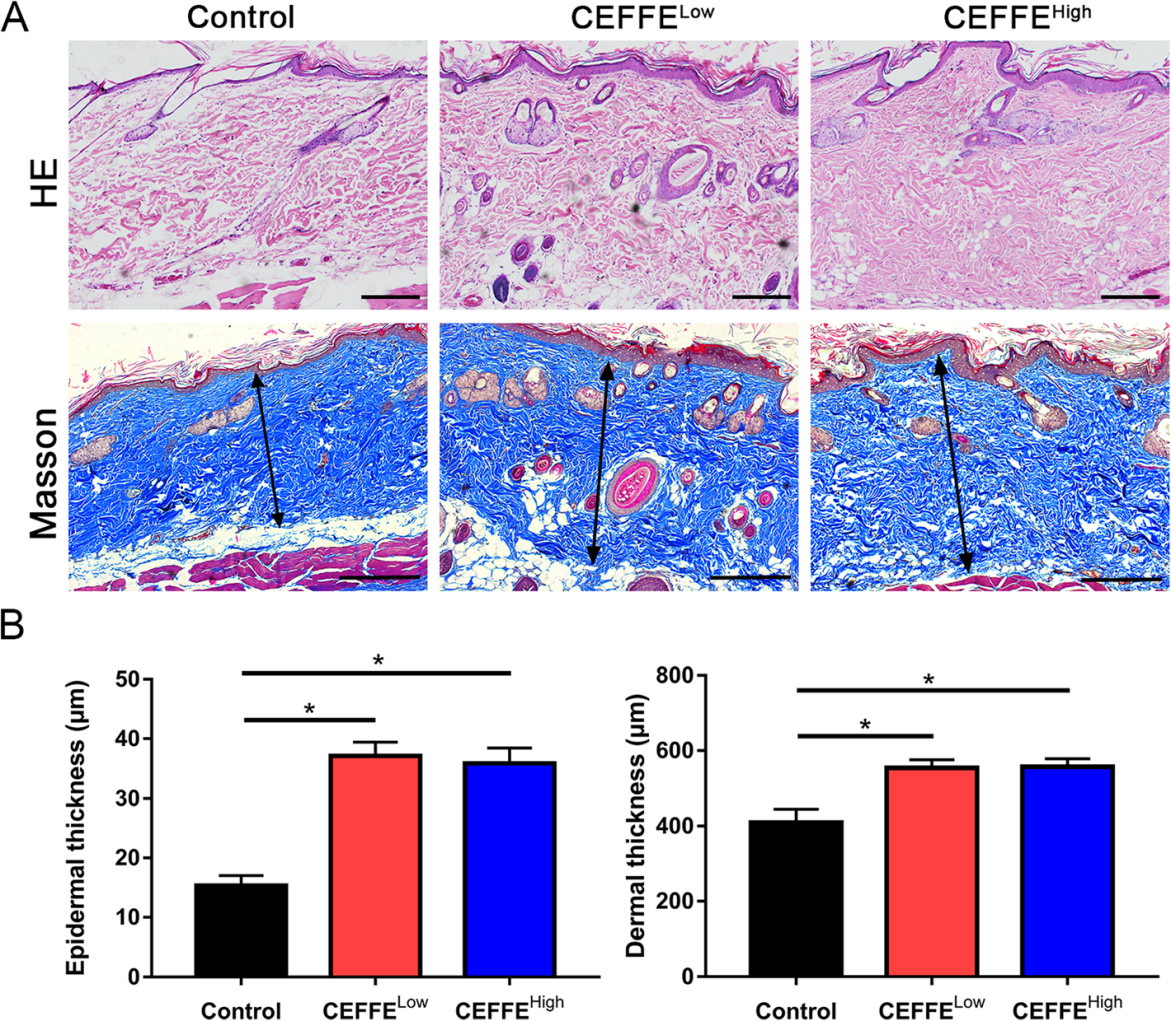
Effect of the cell-free fat extract (CEFFE) on alterations of the epidermis and dermis in the expanded tissue. **a** Hematoxylin and eosin (HE), and Masson’s trichrome staining of expanded tissue. Arrows indicate the dermal portion of the skin. Scale bar: 200 μm. **b** Quantitative analysis of the thickness of expanded tissue. CEFFE treatment significantly increases the epidermal and the dermal skin thickness. Data are expressed as means ± SD. ∗*p* < 0.05

To evaluate the quality of the expanded tissue, histological analyses were performed. The thickness of the epidermis and dermis in the CEFFE-treated group was significantly increased compared with the control group (Fig. 2). In addition, dermal collagen was increased. However, no significant difference was observed between the CEFFE^Low^ and CEFFE^High^ groups.

### CEFFE treatment improved vascularization of the expanded tissue

**Fig. 3:**
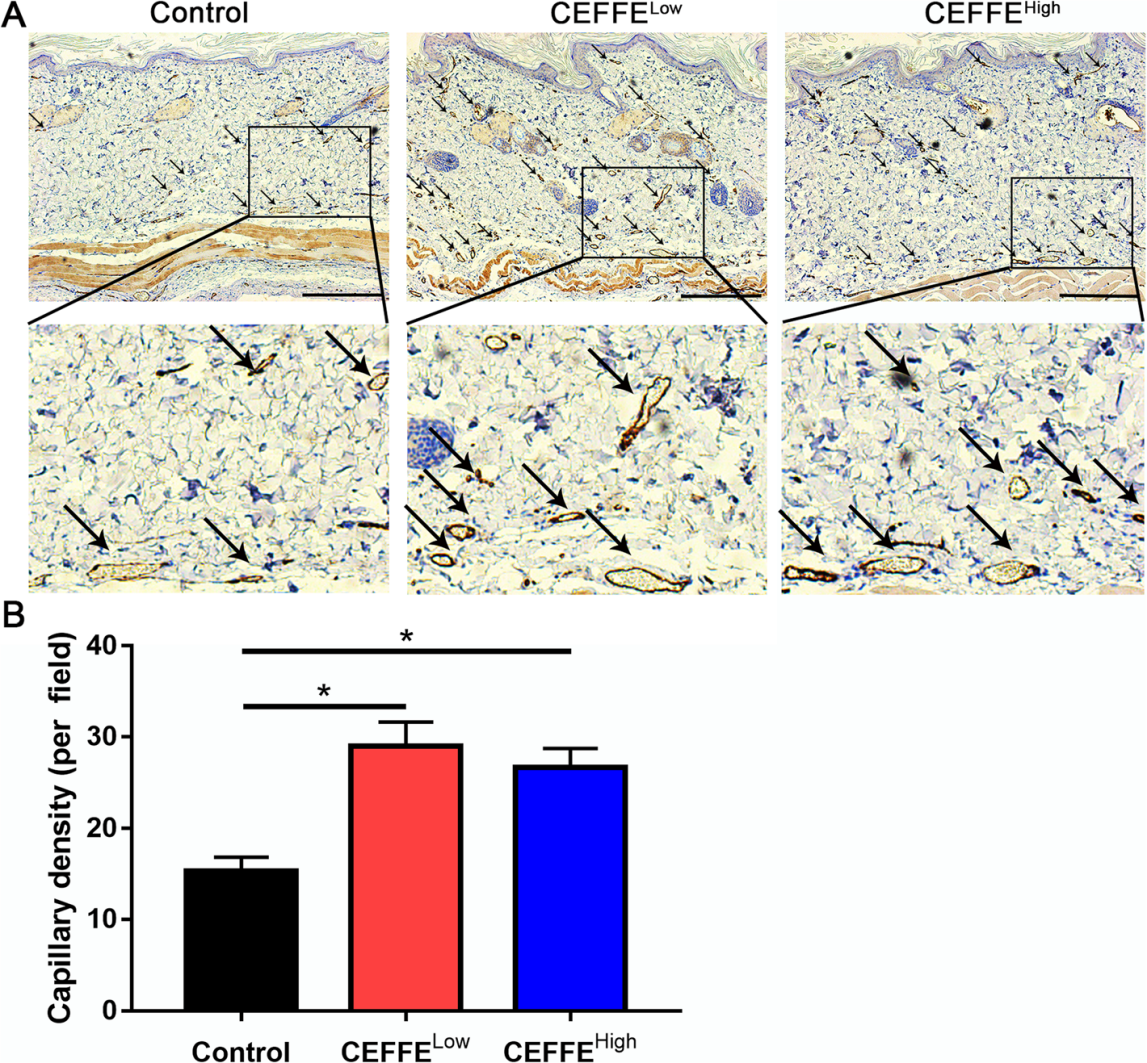
Effect of the cell-free fat extract (CEFFE) on vascularization of the expanded tissue. **a** Immunostaining of CD31 in expanded tissue. Arrows indicate blood vessels. More blood vessels are present in CEFFE-treated tissue. Scale bar: 200 μm. **b** Quantitative analysis of capillary density. CEFFE treatment significantly increases the blood vessel density. Data are expressed as means ± SD. ∗*p* < 0 05

Vascularization plays a key role in tissue survival. We performed anti-CD31 staining to detect the density of blood vessels. As shown in Fig. 3, the number of blood vessels in the CEFFE-treated groups was significantly increased compared with the control group. Again, no significant difference was observed between the CEFFE^Low^ and CEFFE^High^ groups.

### CEFFE treatment increased epidermal cell proliferation

**Fig. 4:**
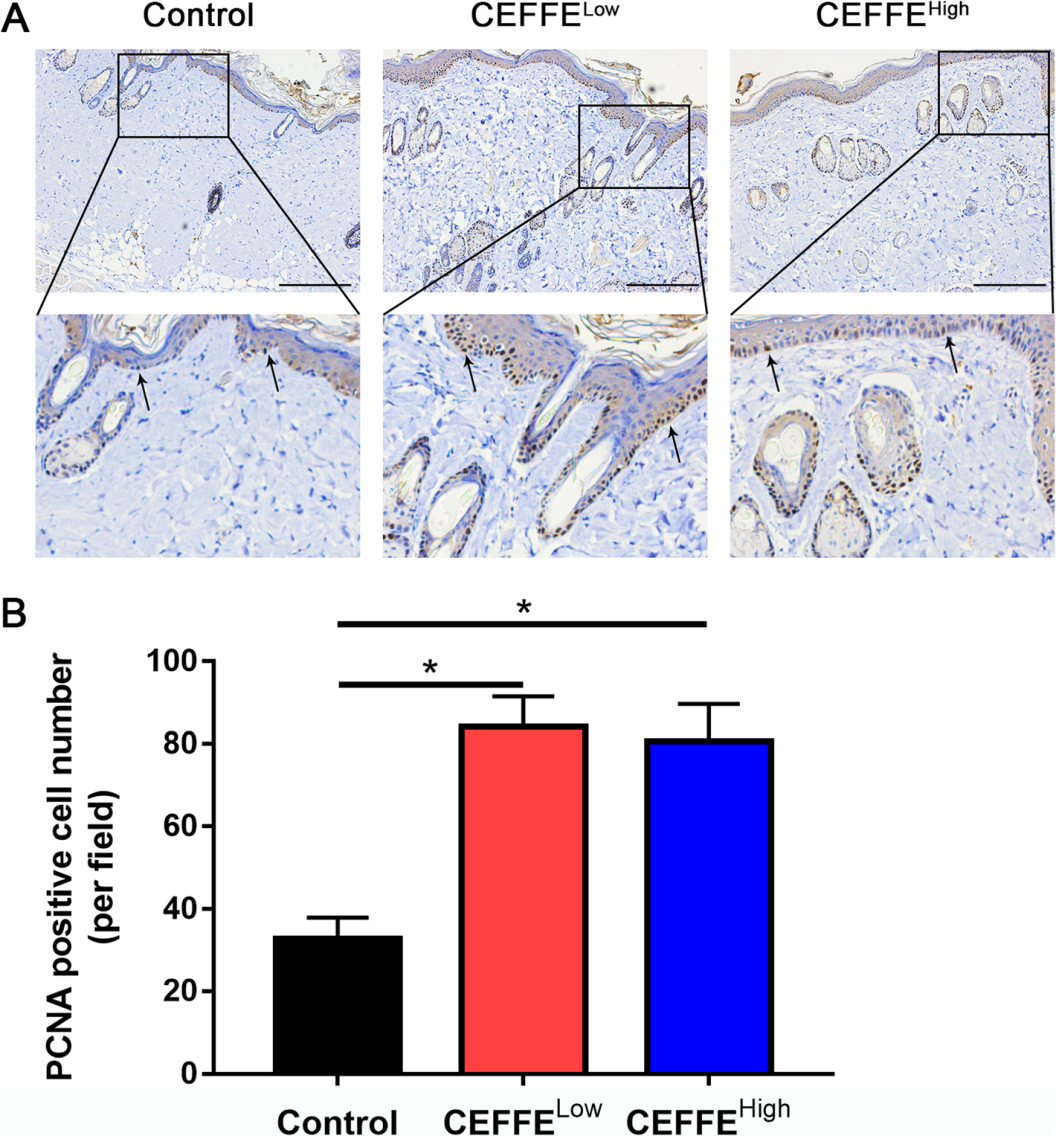
Effect of the cell-free fat extract (CEFFE) on epidermal cell proliferation. **a** Proliferating cell nuclear antigen (PCNA) staining in expanded tissue. Arrows indicate PCNA-positive cells. More PCNA-positive cells are present in CEFFE-treated tissue. Scale bar: 200 μm. **b** Quantitative analysis of the PCNA-positive cell number. CEFFE treatment significantly increases epidermal cell proliferation. Data are expressed as means ± SD. ∗*p* < 0 05

Cell proliferation can promote the growth of the new skin, leading to decreased retraction. PCNA staining was performed to observe cell proliferation in the expanded tissue. The number of PCNA-positive cells in the CEFFE-treated groups was significantly increased compared with the control group, and these cells were mostly located in the epidermal basal layer (Fig. 4). There was no significant difference between the CEFFE^Low^ and CEFFE^High^ groups.

### Western blot analysis of the expanded tissue

**Fig. 5:**
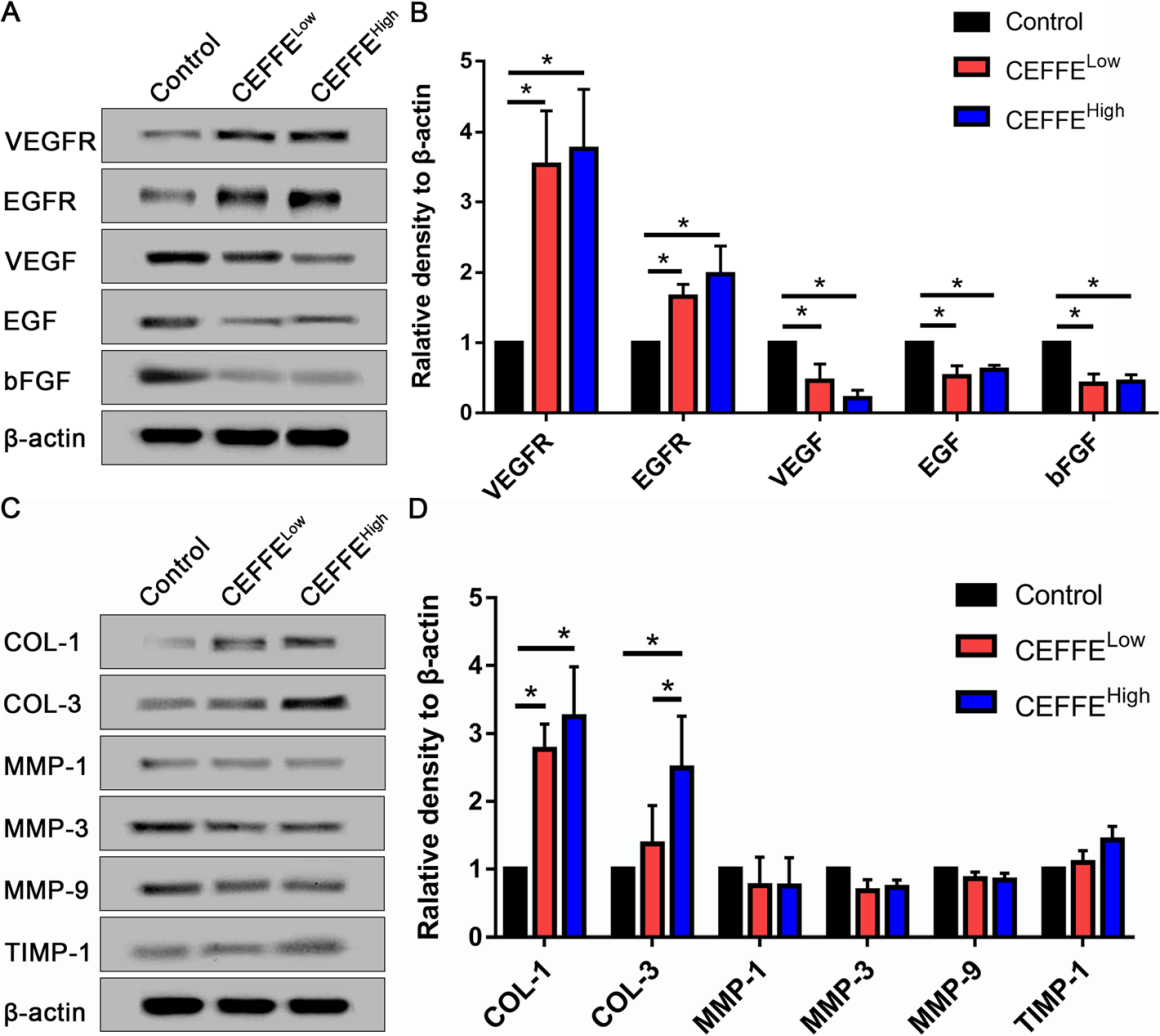
**a**, **b** Western blotting analyses of the vascular endothelial cell growth factor receptor (VEGFR), epidermal growth factor receptor (EGFR), VEGF, EGF, collagen type 1 (COL-1), collagen type 3 (COL-3), and basic fibroblast growth factor (bFGF) in expanded tissue. **a** Representative western blot results from three independent experiments are shown. **b** Cell-free fat extract (CEFFE) treatment significantly increases the expression of VEGFR, EGFR, COL-1, and COL-3. The expression of VEGF, EGF, and bFGF is decreased by CEFFE in the expanded tissue. Density values are expressed in arbitrary units. **c**, **d** Western blotting analysis of COL-1; COL-3; matrix metalloproteinase (MMP)-1, − 3, and – 9; and tissue inhibitor of metalloproteinases 1 (TIMP-1) in the expanded tissue. **c** Representative results from three independent experiments are shown. β-actin was used as the loading control. **d** Density values are expressed in arbitrary units relative to β-actin. Data are expressed as means ± SD. *p < 0.05

To elucidate the mechanism of CEFFE, we performed western blot analyses to analyze the expression of selected proteins in the expanded tissue. Compared with the control group, the expression of VEGFR and EGFR was increased in the CEFFE-treated groups, while VEGF, EGF, and bFGF were decreased (Fig. 5). The expression of COL-1 and COL-3 was increased, but the expression of MMP-1, − 3, and − 9, and TIMP-1 was not significantly changed. These findings indicated that the effect of CEFFE on collagen metabolism mostly involved collagen synthesis, not collagen degradation.

### CEFFE promoted epidermal cell proliferation in vitro

**Fig. 6:**
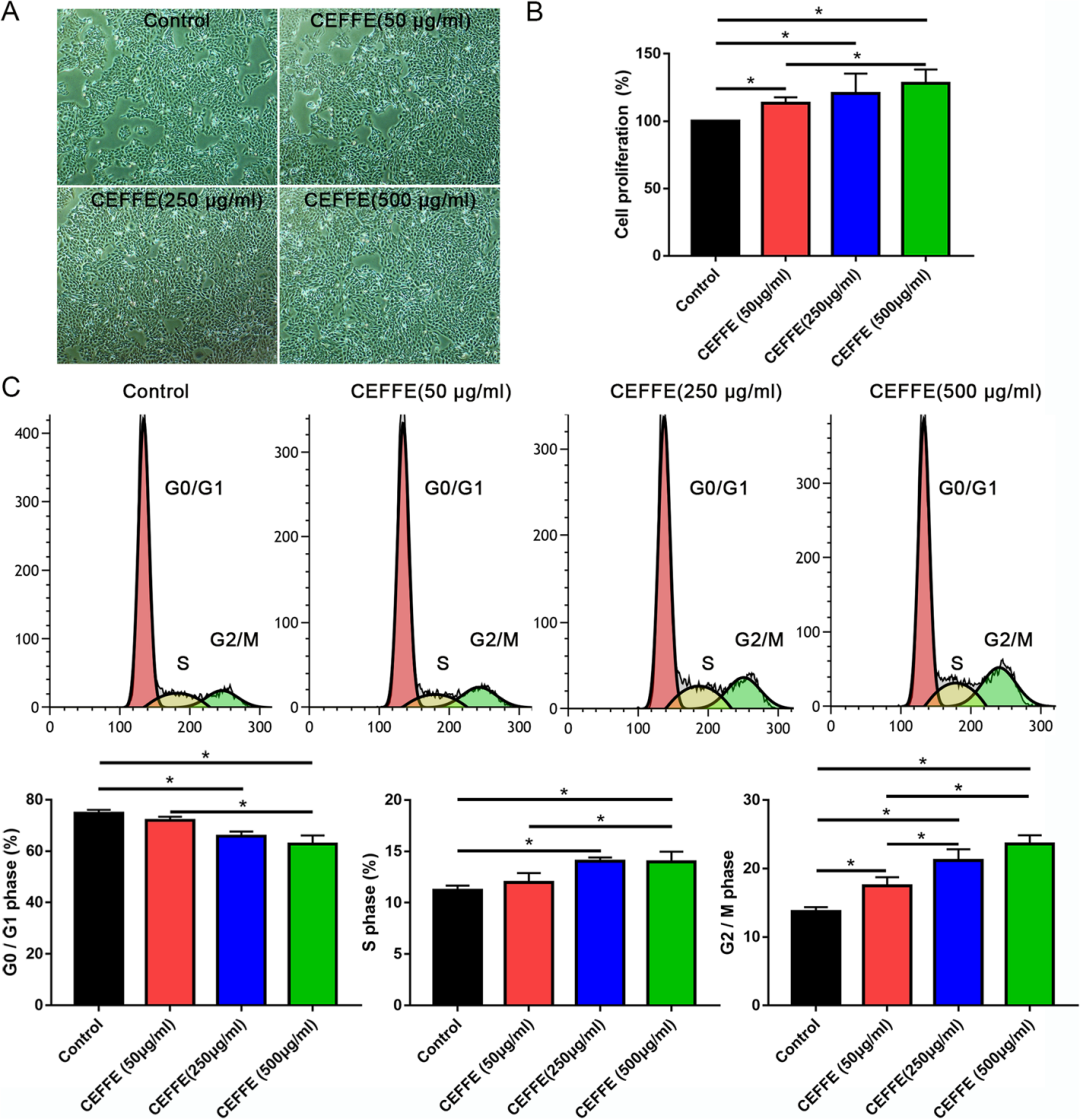
The cell-free fat extract (CEFFE) increases the proliferation of HaCaT cells. **a** Morphology of HaCaT cells 72 h post-CEFFE treatment. Scale bar: 150 μm. **b** The Cell Counting Kit-8 assay shows cell proliferation at 72-h post-CEFFE treatment. **c** Cell cycle distribution analyzed by flow cytometry. Data are expressed as means ± SD. *p < 0.05

Consistent with the in vivo experiments, HaCaT cells treated with different concentrations of CEFFE showed increased cell proliferation. In the CEFFE-treated groups, the proliferation of HaCaT cells was significantly increased in a concentration-dependent manner, compared with the control group (Fig. 6). The cell cycle analysis revealed an increased number of cells in the S phase and G2/M phase in the CEFFE-treated groups.

## Discussion

Tissue expansion techniques play an important role in skin reconstruction [27–29]. These techniques have the advantages of generating high-quality extra skin with well-matched color and texture to the surrounding skin and can be used to repair soft tissue deficiency without leaving another wound on the donor [30, 31]. Working through a simple mechanical stretching to stimulate cell proliferation and tissue regeneration, the expansion process requires a long period of time. In addition, excessive pressure stimulation can lead to tissue necrosis due to an insufficient blood supply. Furthermore, post-operative skin retraction normally occurs when tissue regeneration is insufficient [30, 32]. In the current study, we demonstrated that CEFFE could promote tissue regeneration during tissue expansion. CEFFE treatment was able to reduce the extent of tissue necrosis and retraction (Fig. 1) and increase skin thickness (Fig. 2).

Vascularization is crucial for tissue expansion. Mechanical stretching stimulates new blood vessel formation that attracts essential nutrients as well as cells for tissue regeneration [33]. The speed of tissue expansion is limited by the rate of neovascularization. When the blood supply cannot meet the nutrient and oxygen requirement of the expanded tissue, tissue necrosis normally occurs [17].

Pro-angiogenic strategies are helpful during tissue expansion [17, 34]. Our previous studies demonstrated that CEFFE possesses strong pro-angiogenic activity. CEFFE contains about 59 proteins related to angiogenesis, including VEGF and EGF [25]. It attenuates ischemic injury in a mouse hindlimb ischemic model [25] and improves fat graft [35] and skin flap [36] survival by accelerating blood vessel formation. In the current study, we confirmed that CEFFE promoted angiogenesis during tissue expansion (Fig. 3). The presence of large amounts of pro-angiogenic growth factors in CEFFE likely inhibited the intrinsic expression of VEGF and EGF, but increased the expression of VEGFR and EGFR (Fig. 5), resulting in enhanced blood vessel formation.

Tissue retraction normally occurs when the expander is removed. To reduce the retraction rate of the expanded skin, promoting cell proliferation and the secretion of extracellular matrix in the expanded skin are of vital importance. Mechanical stretching can stimulate the migration and proliferation of adjacent dermal fibroblasts during expansion [13]. Our previous studies found that CEFFE promoted the proliferation of dermal fibroblasts and enhanced the expression of extracellular matrix [26]. In the current study, CEFFE promoted the proliferation of epidermal as well as basal layer cells (Figs. 4 and 6). In addition, CEFFE treatment promoted collagen secretion, which was consistent with previous studies [26]. These effects are likely due to the presence of multiple growth factors like EGF, insulin-like growth factor-1, and bFGF [37, 38] because studies show that the proliferation of epidermal cells and fibroblasts is promoted by EGF [39], and EGF also regulates the expression of collagen [40]. Compared with the control group, CEFFE treatment significantly increased the thickness of the epidermis and dermis, indicating that it could improve the quality of the expanded skin.

In this study, the low-dose (300 μg of total protein) and high-dose (600 μg of total protein) CEFFE treatment groups were compared. There was no significant difference in the treatment efficacy between these two groups, indicating that a low dose of CEFFE is sufficient to support tissue expansion. Recent studies have shown that mesenchymal stem cells can benefit tissue expansion [17]. Compared with cell therapies, CEFFE is a cell-free liquid that avoids the safety issues associated with cell-based therapies. Furthermore, the storage of CEFFE is convenient and multiple treatments are feasible. Because it is cell-free, no immune rejection would occur when allogeneic CEFFE is used. Theoretically, CEFFE could be used as an “off the shelf” material. However, there are still problems need to be solved. As an off the shelf product, the quality control is a basic requirement. It is still unknown which components play a crucial role in CEFFE. Thus, it is difficult to set a standard to achieve a quality control. The functional fraction of CEFFE needs to be dissected in future.

In the current study, all CEFFE are derived from healthy young women. It is unclear whether there is any difference of therapeutic effect between CEFFE from different donors. The effects and components of CEFFE from elderly donors, donors with diabetes, or male donors are worth to be studied in the future. In addition, CEFFE contains a large number of growth factors, such as brain-derived neurotrophic factor, glial cell-derived neurotrophic factor, and neurotrophin-3 [25], that will benefit the tissue expansion. The effects of CEFFE on other aspects of tissue regeneration remain to be further explored.

## Conclusions

In summary, by promoting angiogenesis and cell proliferation, CEFFE could assist the rapid expansion of the skin, improving its quality. Because of these beneficial effects, CEFFE could be potentially used in the clinical application of tissue expansion.

## Data Availability

The data used to support the findings of this study are included within the article.

## Abbreviations

bFGF: Fibroblast growth factor
CEFFE: Cell-free fat extract
COL-1: Collagen type 1
COL-3: Collagen type 3
EGF: Epidermal growth factor
EGFR: Epidermal growth factor receptor
HE: Hematoxylin and eosin
MMP-1: Matrix metalloproteinase 1
MMP-3: Matrix metalloproteinase 3
MMP-9: Matrix metalloproteinase 9
PCNA: Proliferating cell nuclear antigen
TIMP-1: Tissue inhibitor of metalloproteinases 1
VEGF: Vascular endothelial growth factor
VEGFR: Vascular endothelial growth factor receptor
